# Research Priorities for Fertility and Conception Research as Identified by Multidisciplinary Health Care Practitioners and Researchers

**DOI:** 10.3390/nu8010035

**Published:** 2016-01-13

**Authors:** Lisa J. Moran, Laura Spencer, Darryl L. Russell, Mary Louise Hull, Sarah A. Robertson, Tamara J. Varcoe, Michael J. Davies, Hannah M. Brown, Raymond J. Rodgers, Robinson Research Institute Consortium of Fertility and Conception Practitioners

**Affiliations:** 1 The Robinson Research Institute, University of Adelaide, 55 King William Road, North Adelaide, South Australia 5006, Australia; lisa.moran@adelaide.edu.au (L.J.M.); laura.spencer@adelaide.edu.au (L.S.); Darryl.russell@adelaide.edu.au (D.L.R.); louise.hull@adelaide.edu.au (M.L.H.); sarah.robertson@adelaide.edu.au (S.A.R.); tamara.varcoe@adelaide.edu.au (T.J.V.); Michael.davies@adelaide.edu.au (M.J.D.); hannah.brown@adelaide.edu.au (H.M.B.); 2 Monash Centre for Health Research Implementation, School of Public Health and Preventative Medicine, Monash University, Melbourne 3004, Australia; 3 The Robinson Research Institute, University of Adelaide, 55 King William Road, North Adelaide, South Australia 5006, Australia

**Keywords:** preconception, fertility, awareness, nutrition

## Abstract

The Robinson Research Institute of the University of Adelaide convened a multidisciplinary group of *n* = 33 clinicians, researchers and representatives of government organisations on the 2 October 2014 for a workshop entitled “*Promoting fertility and healthy conception. How do we generate greater reproductive health awareness?*” The key aim of the workshop was to assess the body of knowledge that informs clinical practice and government policy, and to identify questions and additional information needed by health practitioners and government representatives working in the field of reproductive health and to frame future research and policy. The workshop identified topics that fell mostly into three categories: lifestyle-related, societal and biological factors. The lifestyle topics included nutrition and diet, exercise, obesity, shift work and other factors deemed to be modifiable at the level of the individual. The societal topics included discussions of matters that are structural, and resistant to change by individuals, including specific ethical issues, social disadvantage, government and educational policies. The biological factors are intrinsic physical states of the individual, and included many factors where there is a dense body of scientific knowledge which may not be readily accessible in less academic language. This workshop thus provided an opportunity to identify further actions that could be undertaken to meet the needs of diverse organisations and groups of professionals with an interest in human fertility. Since so many factors in our social and biological environment can impact fertility and preconception health, it is imperative to involve many disciplines or levels of government or societal organisations that have not traditionally been involved in this area.

## 1. Introduction

Optimising preconception care is recognised as a crucial health priority with regards to improving fertility, maternal obstetric outcomes and fetal, infant and potentially adult health. It consists of the screening, prevention and management of maternal and paternal risk factors that can affect maternal or offspring health. These include behaviours related to lifestyle factors such as weight, diet and supplement use, physical activity, smoking, alcohol or stress management. They also include behaviours related to non-lifestyle aspects such as immunisation, occupational or environmental hazard assessment, consideration of genetic risk factors, screening and management of medical diseases related to potential adverse maternal or child outcomes, review of medications and consideration of age [1]. Preconception care is being increasingly incorporated into position statements or guidelines worldwide [1,2,3,4]. The scope of these guidelines varies with preconception recommendations for women with chronic diseases being consistently provided.

Information on modifiable lifestyle factors is provided less consistently in position statements and guidelines and often varies from country to country [1]. Many people therefore may not receive appropriate preconception guidance to allow them to choose healthy lifestyle behaviours or preconception health habits. This is supported by a representative sample of reproductive-aged Australians having poor knowledge of both modifiable (e.g., obesity and smoking) and non-modifiable (e.g., age) factors associated with fertility [5]. This highlights both the importance of awareness of appropriate preconception health and the implementation of clinical care to support optimal preconception health.

There is a need for identification of common clinical and research priorities to enhance preconception clinical care. The aim of this is to lead to increased consistency in health care, research collaboration, community engagement and government interaction in this important clinical sphere. With this in mind, the Robinson Research Institute, a leading Australian clinical and research institute in human reproduction, pregnancy and child health, convened a workshop with the aim of assessing the key clinical needs of health practitioners and government representatives working in the field of reproductive medicine to identify research priorities to meet their needs and those of their clients.

## 2. Experimental Section

### 2.1. Participants

The Robinson Research Institute, University of Adelaide convened a multidisciplinary group of *n* = 33 clinicians, researchers and government representatives in the evening of 2 October 2014 for a workshop entitled “*Promoting fertility and healthy conception. How do we generate greater reproductive health awareness?*” The aim of the workshop was to assess the clinical and research priorities of health practitioners, researchers and government representatives working in the field of reproductive medicine. The attendees included representatives of the Robinson Research Institute (specifically the authors of this article LJM, LS, DLR, MLH, SAR, TJV, MJD, HMB and RJR) with backgrounds including basic science, epidemiology, dietetics and clinical treatment of infertility. Members of the Robinson Research Institute Fertility and Conception Practitioners Consortium (see Supplementary information) with a diverse range of backgrounds and interest in this topic also participated. These included diabetes educators, dietitians, psychologists, nurses, naturopaths, clinicians and scientists. Additional invited guests from Victorian Assisted Reproductive Treatment Authority (VARTA) which is an Australian State government organisation that provides independent information and support for individuals, couples and health professionals on fertility and issues related to assisted reproductive treatment [6]. The aim of the workshop was to include a range of disciplines including allied health practitioners, medical health practitioners, researchers and the government.

### 2.2. Workshop

The workshop was facilitated by Adrianne Pope a professional facilitator with an extensive background in the IVF industry including as senior manager of Monash IVF, president of the Fertility Society of Australia and board member of VARTA. She discussed understanding stakeholder needs, identifying key research questions and developing clinical networks or partnerships. The attendees were then addressed by VARTA with their perspective on issues surrounding human fertility and conception in the assisted reproductive technology area. They explained that VARTA was a statutory authority providing independent information and support for individuals, couples and health professionals on fertility and issues related to assisted reproductive treatment and monitoring developments, trends and activities relating to the causes and prevention of infertility and the assisted reproductive treatment industry. VARTA have invested considerable resources into online education and information.

The key discussions initiated by the facilitator were on engaging clinical partners in research, with the objectives of understanding the stakeholders’ needs and identifying key research questions. Attendees were arranged in small groups composed of *n* = 5–8 people of different backgrounds and interests with each group containing at least one representative of the Robinson Research Institute with an active role in research to aid group facilitation. Following small group discussions the comments from each group were compiled and presented back to the whole group for further consideration and discussion. The results were then summarised and described narratively.

## 3. Results

The key themes identified by the workshop participants included lifestyle-related factors, social and biological factors related to preconception health and fertility (Table 1, Table 2 and Table 3). With regards to lifestyle factors, the effect of diet on fertility, the issue of identifying specific diets or nutrients that could be recommend preconception or that could be tailored to individuals were discussed. In addition to this, other key points included aiding people to make these dietary changes and prescribed and increasing motivation. The adverse effect of obesity and other chronic diseases such as heart disease and cancer on fertility, the impact of the epidemics of these chronic lifestyle-related diseases on resource utilisation and the impact of other chronic diseases such as PCOS and endometriosis on fertility and lifestyle factors were raised. While these were discussed predominantly in the context of female health, their potential impact on male fertility was also highlighted as being necessary to understand. The key role of allied health professionals such as dietitians in providing preconception lifestyle management was highlighted. The adverse role of stress on fertility and the role psychologists in identification and management of stress in a preconception environment was discussed. Other lifestyle issues discussed included the importance of appropriate sleep patterns on fertility (Table 1).

**Table 1:** Lifestyle-related factors and preconception health and fertility.

How can we assist in motivating people to make lifestyle changes preconception?
Identify the impact of micronutrients on fertility and live birth
Can diets be tailored to specific individuals to aid in changing lifestyle?
How does diet affect fertility?
What is the next epidemic wave in the developing world? Obesity; heart disease; cancer; diabetes? How will this impact fertility, pregnancy and life expectancy?
With the obesity epidemic, are there sufficient resources to address the problem? What skills are required?
What factors affect male fertility? What are the drivers of male infertility?
Would public reimbursement of treatments for lifestyle issues aid in improving general health?
What can be done to utilise positive reinforcement messages with people trying to make lifestyle changes?
How are allied healthcare providers, such as personal trainers, included in the loop of fertility care?
What role does stress play in fertility? How can it be measured and managed?
Does infertility impact mental health? If so, how and how might this affect preconception health?
What role does diabetes play in fertility?
What effect does shift work or disturbed sleep patterns have on fertility?
How can women be diagnosed for endometriosis/PCOS and educated on the fertility, lifestyle and health issues associated with these diseases at a young age?

The societal and social issues that impact fertility and preconception health were also raised by the participants (Table 2). These included identifying specific cultural or religious barriers to family planning and the effects that social and economic status and political issues could have on fertility. Education and government initiatives were highlighted as being a possible conduit to the population for messages around reproductive health. One initiative considered government funding of individuals for maintenance of a good lifestyle conductive to good reproductive health. Gender differences in how messages of these topics are perceived was also discussed and identified, with comments about how we can improve our engagement with men. New emerging technologies and the speed at which they can be implemented were considered an issue for noting.

**Table 2:** Societal factors and preconception health and fertility.

Explore the social, economical and political issues that impact fertility
What are the cultural barriers in family planning? Could these be altered?
How can people be educated about their fertility? How should the messages be delivered and by whom? Should they be specifically directed by gender? Do we understand communication?
What role does education play in preparing young people for fertility issues throughout life and how can primary healthcare providers assist?
How can government policy be altered to reflect supporting reproduction at an earlier age?
How should research be planned to address the speed at which new technology is introduced without ample supportive evidence?
Would public reimbursement of treatments for lifestyle issues aid in improving general health?

A large number of biological factors that affect fertility and preconception health were raised for consideration (Table 3). Whilst clearly some have been investigated scientifically, clinically or epidemiologically, the fact they were raised for discussion indicates that either more investigation is needed or that a consensus does not exist or that the messages around these are not coherent enough at this stage. Some of the more interesting questions included the question as to why humans are such poor reproducers when compared to other animals. Of interest was why oocytes age, can this be prevented or reversed and additionally what is the cause of menopause. Other discussions identified many areas that we know can affect preconception health and fertility where it is not exactly clear how these occur and even what the severity of their impact might be.

**Table 3:** Biological factors and preconception health and fertility.

Understand the endocrine pathways in fertility. What are the effects of endocrine disrupters on fertility?
Identify which major environmental factors have an impact on fertility
What makes a “good” egg with potential to generate an embryo, pregnancy and healthy birth?
What are the origins of infertility? Has it developed over time? Is it a chronic disease?
What are the oocyte-related mechanisms and consequences of oocytes in hostile environments? Are there repair mechanisms?
Does endometriosis affect fertility? If so, how?
What is the impact of *in vitro* fertilisation? Do outcomes reflect the patient or the actual technology?
Long term trans generation studies must be undertaken to assess the safety and health impact on children born from assisted reproductive technology.
Do chronic diseases have an impact on fertility? Specifically, do auto immune diseases alter fertility?
How are pregnancy outcomes assessed and children’s or adult’s health monitored long-term?
What impact does infertility and treatment have on future generations?
How can early pregnancy be monitored and what are the events of conception? How can the actual mechanisms be determined?
Why are humans such poor reproducers? Has this changed over time?
How can the aetiology of infertility be elucidated?
What causes fertility to decline in women?
Can folliculogenesis be slowed to extend delay menopause?
As age is recognised as a limiting factor in fertility, how can the reproductive life be lengthened for females and males?
How can ageing of oocytes be reversed? What is the mechanism for aneuploidy and could it be prevented?
What causes menopause? Why do some woman have significantly shorter reproductive lives?
Can damage from autoimmune diseases be prevented?
What are the mechanisms of implantation? What role does the immune system play in implantation?

## 4. Discussion

We report here on the clinical and research priorities highlighted by a group of multidisciplinary clinicians and researchers on the theme of preconception care and fertility. We report broadly on lifestyle-related, societal and biological factors that were identified as needing further consideration.

The issue of the optimal preconception diet and the best means of achieving this diet were raised as high priorities. There is increasing research examining the effect of preconception nutrition on outcomes including fertility and pregnancy and child outcomes. Dietary factors including lower dietary glycaemic load [7], lower animal protein [8], improved fatty acid profile (decreased trans or saturated fats or increased omega 3 intake) [9,10,11] or greater adherence to a Mediterranean-type dietary pattern [12,13] are associated with lower risk of difficulty in getting pregnant, a decreased risk of ovulatory infertility and an increased probability of getting pregnant or increased oocyte number or improved embryo morphology following IVF/ICSI. While not specifically raised at the workshop, the association of the status of micronutrients such as vitamin D with reproductive health is also an area of interest given the potential association of vitamin D with infertility, ART outcomes, PCOS, endometriosis, pregnancy outcomes and sperm quality [14,15].

The role of allied health professionals including dietitians, exercise physiologists and psychologists in providing education and guidance on achieving optimal preconception health behaviours was also highlighted. Their role is key in optimising preconception health given the health implications of excess weight on infertility and adverse obstetric and fetal outcomes [16], the increasing prevalence of overweight and obesity in women entering pregnancy [17,18,19,20] and the need for optimising diet and physical activity behaviours for prevention and management of overweight and obesity [21]. The role of stress preconception or during assisted reproduction is also recognised as being potentially deleterious to fertility and should be appropriately identified and managed [22]. Given the recognised difficulties in long-term sustainability to healthy lifestyle programs, particularly in young adults [23] there is also a need to identify models of clinical care whereby these recommendations can be made. This will involve consideration of the best means of improving motivation and adherence to healthy lifestyle messages in a clinical environment [24].

International priorities in preconception health include the need for collaborative research to develop consistent evidence-based guidelines for preconception health and care [1]. The model of clinical care whereby preconception care services are provided also varies from country to country and can range from opportunistic assessment by general practitioners or family planning clinics to standardised gynaecological care of high risk women [1]. This is likely to lead to different challenges to implementation of care. For example, a qualitative study examining preconception care practices of general practitioners highlighted factors including time constraints, competing priorities, a lack of women presenting preconception and a lack of preconception specific resources [25]. This needs to be examined in country-specific settings to aid the implementation of preconception recommendations. Large scale implementation of preconception guidelines is possible, with a recent study from Shanghai, China reporting that a free preconception care program to prevent birth defects increased awareness of and participation in preconception care in both men (90%) and women (87%) [26].

The issue of societal barriers to fertility was also considered a high priority. For example, cultural variability in the desire for a high level of female fertility contributes to social stigma and creates an imperative for clinical interventions, such as IVF. People from low socioeconomic status or those living in remote areas or in indigenous communities are at risk of poor reproductive health for many reasons, including lack of timely and appropriate access to medical care, high rates of infection, poor housing and substance abuse, such as smoking. Gender was also identified as a major issue with younger men unlikely to heed messages or unlikely to consider the messages applicable to them [27]. Of interest, young people at the latter years of schooling were identified as a key group whereby preconception health messages could first be targeted to raise awareness to allow early engagement with positive lifestyle messages [28]. However, we note that high school teachers were invited to the workshop but none attended. This is a particularly important group of people who should therefore be included in future discussions on preconception health priorities.

A large number of biological factors that affect fertility and preconception health were also raised for consideration. Many of these have been reported on and discussed in the professional literature. While not all the areas identified are ready to be translated into recommendations for preconception health, institutions involving multidisciplinary and collaborative and translation research and engagement with external professionals such as the Robinson Research Institute could contribute through methods such as production of education materials for informing consumers and health professionals. Of note, The Robinson Research Institute would be in a key position to facilitate this due to its strong basic science, clinical and translation in this area including substantial contribution to the first evidence based guidelines for management of PCOS [29]. Members of the Robinson Research Institute are also involved at senior positions in professional societies, government and clinical organisations, such as VARTA and the Jean Hailes Foundation, and with major funding bodies.

## 5. Conclusions

The workshop identified many areas requiring attention and identified opportunities for the Robinson Research Institute to partner with other organisations and diverse groups of professionals with an interest in this area. In the time since this workshop was conducted, research has commenced in the identified priorities with publications by the listed authors [30,31,32,33,34,35,36]. There is a need for clearer concise quality information that bridges all areas of knowledge around many sub topics in this area. There is also a need for additional evidence-based guidelines that present the evidence, weigh it accordingly and make recommendations for treatment and improvement of lifestyle. Since so many factors in our social and biological environment can impact fertility and preconception health, it will be imperative to involve many disciplines, levels of government or societal organisations, that traditionally have not been involved in this area.

## Supplementary Files

Supplementary File 1
