# Supplementary material for: Research Priorities for Fertility and Conception Research as Identified by Multidisciplinary Health Care Practitioners and Researchers
Source: Nutrients. 2016 Jan 13;8(1):35. doi: 10.3390/nu8010035 (PMC4728649; doi:10.3390/nu8010035)
Supplement: Supplementary File 1 [file nutrients-08-00035-s001.docx]

**Supplementary Materials: Research Priorities for Fertility and Conception Research as Identified by Multidisciplinary Health Care Practitioners and Researchers**

Lisa J. Moran, Laura Spencer, Darryl L. Russell, Mary Louise Hull, Sarah A. Robertson, Tamara J. Varcoe, Michael J. Davies, Hannah M. Brown,
Raymond J. Rodgers and Robinson Research Institute Consortium of Fertility and Conception Practitioners

**Table S1.** Members of the Robinson Research Institute and the Consortium of Fertility and Conception Practitioners who attended the workshop listed with their affiliation and major specialty.

| **Name** | **Organisation** | **Specialty** |
| --- | --- | --- |
| Helen Alvino | University of Adelaide | Clinical Research nurse |
| Rhea Bergmann | Cate Howell & Associates | Dietitian |
| Kerryn Boogard | Diabetes SA | Dietitian |
| Hannah Brown | University of Adelaide | Research fellow, embryo Development |
| Michael Davies | University of Adelaide | Research fellow, epidemiologist |
| Jacki Dellavedova | Private practice | Diabetes educator, dietitian |
| Kate Gallasch | Adelaide Community Healthcare Alliance | Dietitian, obstetric and women’s health |
| Karin Hammarberg | Victorian Assisted Reproductive Treatment Authority | Research fellow. Nurse in IVF. |
| Gill Homan | Flinders Fertility | Fertility Nurse Manager |
| Louise Hull | Fertility SA, University of Adelaide | Clinician, Fertility Specialist |
| Louise Johnson | Victorian Assisted Reproductive Treatment Authority | Chief executive officer. Microbiologist |
| Lea Papworth | Lea Papworth Acupuncture | Acupuncture for fertility |
| Leanne March | Adelaide Women’s Health Centre | Clinical Manager, Midwife |
| Kasia Main | Flinders Medical Centre | Dietitian |
| Lisa Martin | Monash University | Academic, chemistry. |
| Angela McLean | Repromed | Clinician, public health medicine; reproductive/fertility |
| Lisa Moran | University of Adelaide | Research fellow, dietitian, obesity in pregnancy |
| Lauren Nevin | Queen Elizabeth Hospital, Woodville | Dietitian |
| Deepti Parmar | Private practice | Dietitian |
| Amina Pearson | Flinders Medical Centre | Dietitian |
| Adrianne Pope | Adrianne Pope Consulting | Consultant in IVF |
| Ray Rodgers | University of Adelaide | Research Fellow, ovarian biology |
| Sarah Robertson | University of Adelaide | Director Robinson Research Institute, research fellow fertility |

**Table S1.** *Cont.*

| **Name** | **Organisation** | **Specialty** |
| --- | --- | --- |
| Darryl Russell | University of Adelaide | Research Fellow, oocyte biology |
| Anne Schneyder | Nutrition Professionals Australia | Dietitian |
| Paula Scanlon | Fertility SA | Unit Nurse Manager |
| Laura Spencer | University of Adelaide | Research, clinical trials |
| Kelton Tremellen | Repromed | Fertility specialist, endocrinologist |
| Kandiah Umapathysivam | Research Fellow, Joanna Briggs Institute | Research scientist, reproductive medicine background |
| Rebecca Urban | Naturopath | Naturopath, specialising in fertility |
| Tamara Varcoe | University of Adelaide | Research fellow, circadian physiologist |
| Samantha White | Adelaide Exercise Physiologist Health Group | Dietitian |
| Deirdre Zander-Fox | Repromed | Clinical Embryologist |
